# Comparison of adjuvant and adjuvant-free murine experimental asthma models

**DOI:** 10.1111/j.1365-2222.2009.03260.x

**Published:** 2009-08

**Authors:** M L Conrad, A Ö Yildirim, S S Sonar, A Kılıç, S Sudowe, M Lunow, R Teich, H Renz, H Garn

**Affiliations:** * Department of Clinical Chemistry and Molecular Diagnostics, Medical Faculty, Philipps University of Marburg, Biomedical Research Center (BMFZ)Marburg, Germany; † Department of Dermatology, Johannes Gutenberg University, Clinical Research Unit AllergologyMainz, Germany; ‡ Department of Experimental Neurology, Medical Faculty, Philipps University of Marburg, Biomedical Research Center (BMFZ)Marburg, Germany

**Keywords:** adjuvant, alum, animal model, BALB/c, behaviour, β-galactosidase, C57BL/6, intraperitoneal, ovalbumin, subcutaneous

## Abstract

**Introduction:**

The most widely used protocol for the induction of experimental allergic airway inflammation in mice involves sensitization by intraperitoneal (i.p.) injections of the antigen ovalbumin (OVA) used in conjunction with the adjuvant aluminium hydroxide (alum). Although adjuvants are frequently used, there are questions regarding the necessity of alum for murine asthma studies due to the non-physiological nature of this chemical.

**Objective:**

The objective of this study was to compare experimental asthma phenotypes between adjuvant and adjuvant-free protocols of murine allergic airway inflammation in an attempt to develop a standardized alternative to adjuvant use.

**Method:**

An adjuvant-free OVA model of experimental asthma was investigated in BALB/c mice using i.p. or subcutaneous (s.c.) sensitization routes. For the s.c. sensitization, β-galactosidase (β-gal) was also tested as an antigen. In addition, OVA adjuvant and adjuvant-free sensitization protocols were compared in BALB/c and C57BL/6 mice. Open-field testing was performed to assess the effect of alum on mouse behaviour.

**Results:**

Comparison of adjuvant vs. adjuvant-free and i.p. vs. s.c. protocols revealed that both adjuvant use and route of antigen application significantly influenced OVA-specific antibody production. Comparison of adjuvant and adjuvant-free protocols in this study clearly demonstrated the non-requirement of alum for the induction of acute allergic airway inflammation, as both protocols induce a similar disease phenotype. BALB/c mice were significantly more susceptible than C57BL/6 mice to sensitization. Using the improved s.c. adjuvant-free protocol, it was demonstrated that alternative antigens such as β-gal can also be utilized. Behavioural studies indicated severe distress in mice treated with alum.

**Conclusion:**

The OVA s.c. adjuvant-free protocol used in this study generates a phenotype comparable to the benchmark adjuvant protocol widely used in the literature. The adjuvant-free alternative avoids the added complication of non-physiological adjuvants that may interfere with asthma treatment or prevention strategies.

## Introduction

Laboratory mice are widely utilized to study a multitude of diseases and are essential for the analysis of *in vivo* mechanisms. With regard to allergic bronchial asthma, mouse models provide an excellent means to examine phenotypes such as lung inflammation, airway reactivity, bronchoalveolar lavage (BAL) cell counts, antigen-specific antibody titres and the expression of inflammatory cytokines.

Several methods are used to induce experimental asthma in the mouse and the most frequently used protocol in the literature involves sensitization by intraperitoneal (i.p.) injections of antigen used in conjunction with the adjuvant aluminium hydroxide (alum) [1]. Although alum is routinely used as an adjuvant, there are several questions regarding the necessity of its use. Alum induces mast cell-independent allergic inflammation; therefore, investigations involving mast cells require an adjuvant-free protocol [2]. In addition to this, as alum is a non-physiological substance with a very recently elucidated mechanism [3–9], it is still unknown as to how this adjuvant may interact with preventative therapeutic agents. Studies examining asthma prevention or treatment strategies would benefit from the use of an adjuvant-free protocol.

In addition to the non-physiological nature of alum, this adjuvant is not required for the generation of an acute allergic inflammatory response using the antigen ovalbumin (OVA) [10]. The literature describes several adjuvant-free protocols for experimental asthma induction including aerosol sensitization via the intranasal (i.n.) [11], intratracheal [12] or exposure chamber routes [13], adjuvant-free parenteral sensitization [14–16] and adoptive transfer of allergen-pulsed T cells [17]. All these protocols generate phenotypes typical of acute allergic inflammation with varying levels of severity; however, exposure to OVA via the aerodigestive route in most cases produces only very weak or no sensitization [13, 18], and usually leads to the induction of mucosal tolerance [19–22]. Parenteral (i.p. or s.c.) adjuvant-free injections of antigen consistently result in strong sensitization, although numerous sensitizations or lengthy challenge times are required. In order to generate an adjuvant-free protocol that can be compared against typical adjuvant protocols, we chose to simplify a parenteral adjuvant-free model by creating a protocol with fewer sensitizations and shorter challenge times.

With the recent resurgence of interest in murine experimental asthma models and the action of adjuvants, it is timely to include adjuvant-free alternatives in the discussion. The objective of this study is to optimize adjuvant-free sensitization with regard to a fully developed phenotype of allergic airway inflammation and clinical features of experimental asthma. To illustrate the effectiveness of the adjuvant-free protocol, comparison of the phenotypes generated by adjuvant and adjuvant-free protocols will be performed in BALB/c and C57BL/6 mice.

## Materials and methods

### Animals

Female BALB/c and C57BL/6 mice aged 6–8 weeks were obtained from Harlan Winkelmann (Borchen, Germany) and housed four animals per cage in a 12/12 h light/dark cycle with food and water available *ad libitum*. All experimental procedures were approved by the local animal ethics committee and met German and international guidelines. At least eight animals were used per group.

### Experimental design

**Fig. 1:**
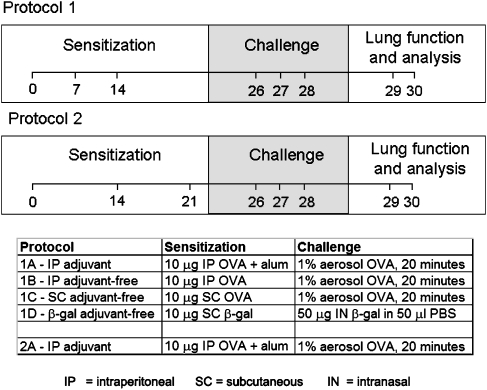
Sensitization and challenge protocols used for murine experimental asthma induction.

Investigations concerning the effect of adjuvant and route of sensitization were all performed using Protocol 1 (see Fig. 1). The i.p. adjuvant protocol, Protocol 1A, consisted of i.p. injections of 10 μg OVA (grade VI) emulsified in 1.5 mg alum (Pierce, Rockford, IL, USA) in 200 μL phosphate-buffered saline (PBS) on days 0, 7 and 14, followed by 20 min 1% OVA (grade V) aerosol treatments on days 26, 27 and 28. The adjuvant-free protocols, Fig. 1– Protocol 1B and 1C, consisted of i.p. or s.c. injections of 10 μg OVA (grade VI; Sigma, Steinheim, Germany) in 200 μL PBS or sham injections of PBS on days 0, 7 and 14. The sensitization phase was followed by 20 min 1% OVA (grade V; Sigma) or sham PBS aerosol treatments on days 26, 27 and 28. s.c. injection was performed at the scruff of the neck and was verified by the observation of a fluid bubble forming under the skin during injection. I.p. injection was performed in the lower right quadrant of the abdomen.

Alternatively, mice were sensitized s.c. with 10 μg β-galactosidase (β-gal) (Sigma) in 200 μL PBS or sham injections of PBS only and i.n. challenged on days 26, 27 and 28 with 50 μg β-gal in 50 μL PBS or sham PBS only (Fig. 1– Protocol 1D). I.n. challenge was chosen with β-gal due to the limited availability of this recombinant antigen in contrast to OVA.

Finally, comparisons of the experimental asthma phenotype generated by adjuvant and adjuvant-free protocols in BALB/c and C57BL/6 mice were performed using the s.c. adjuvant-free and i.p. adjuvant methods, Fig. 1– Protocol 1C and 2A, respectively. Protocol 2A consisted of i.p. injections of 10 μg OVA (grade VI) emulsified in 1.5 mg alum (Pierce) in 200 μL PBS on days 0, 14 and 21, followed by 20 min 1% OVA (grade V) or sham PBS aerosol treatments on days 26, 27 and 28.

#### Airway reactivity

Lung function analysis was performed using non-invasive head-out body plethysmography 24 h after the last aerosol challenge. The mid-expiratory airflow (EF50) of bronchial responsiveness to β-methacholine was measured as described previously [23].

#### Antibody titres

Forty-eight hours after the last aerosol challenge, mice were terminally anaesthetized with ketamine plus rompun and blood samples were taken from the axillary vessels. The levels of OVA-specific IgE and IgG1 were measured by an enzyme-linked immunosorbent assay (ELISA) (BD Bioscience, San Diego, CA, USA) in the serum as described previously [13].

#### Bronchoalveolar lavage

BAL was performed 48 h after the last challenge using 1 mL PBS containing 1 × protease inhibitor cocktail (Roche, Mannheim, Germany) as described in Neuhaus-Steinmetz et al. [24]. An automated Casy TT cell counter (Schaerfe Systems, Reutlingen, Germany) was used to determine the total leukocyte cell counts. Cells were centrifuged and the cell-free supernatant was stored at −20 °C until cytokines were measured by ELISA. For differential cell counts, cytospin preparations were fixed and then stained with Diff-Quick (Merz & Dade AG, Dudingen, Switzerland). Macrophages, lymphocytes, eosinophils and neutrophils were identified by standard morphologic criteria and 300 cells counted per cytospin.

#### Measurement of cytokines

Cell-free supernatants from BAL fluids were analysed for IL-5, IL-10, IL-13, TNF-α and IFN-γ content by sandwich ELISA as described previously in Herz et al. [25]. The detection limit of each ELISA was 10 pg/mL.

### Histology of the airways

Directly after BAL, lungs were fixed with 10% formalin via the trachea, removed and stored in 10% formalin. Lung tissues were embedded into paraffin and 3 μm sections were stained with haematoxylin and eosin (HE) or periodic acid Schiff (PAS).

### Behavioural studies

To determine the effect of alum on mouse behaviour, BALB/c mice were tested using an open-field test [26]. Animals were either left untreated or injected with a sham (PBS) or an adjuvant (alum+PBS). After treatment, animals were allowed a 15-min, a 2- or 4-h rest period, and then subjected to open-field testing for 15 min. The open field consisted of a 41 × 41 × 40 cm black acrylic box monitored by an automated activity system (TruScan™, Photobeam Sensor-E63-22, Coulbourn Instruments, Whitehall, PA, USA). Measures of average velocity (cm/s) and rearing behaviour (number of rears) were taken. Video analysis of the data was conducted with the Bioserve Viewer II Software video tracking system, with infrared beams to measure the rearing activity (http://www.biobserve.com).

### Statistical analysis

Graphing and statistical analysis of normally distributed data was performed using Prism 5 (Graph Pad Software, San Diego, CA, USA). Data are expressed as mean ± SEM and are analysed for significance using Student's *T*-test (in the case of a two group comparison), one-way anova with Tukey's Multiple Comparison Test (for multiple group comparison) or two-way anova for comparison of multiple groups with two influencing factors (protocol vs. strain).

## Results

### Allergic sensitization in mice – differences due to adjuvant use and site of sensitization

Using Protocols 1A and 1B (Fig. 1), an initial experiment was conducted with BALB/c mice and the antigen OVA to determine the contribution of adjuvant to allergic sensitization. Mice sensitized i.p. with the adjuvant alum expressed significantly higher levels of OVA-specific IgG1 in the serum than mice sensitized i.p. adjuvant-free. No differences were observed in the OVA-specific IgE titres; Table 1.

**Table 1:** The effect of adjuvant use and route of sensitization on OVA-specific antibody titres in BALB/c mice

	IP adjuvant	IP adjuvant-free	SC adjuvant-free
Group	Protocol 1A	Protocol 1B	Protocol 1C
OVA-specific IgE (ng/mL)	67 ± 11	41 ± 3.6	110 ± 19***
OVA-specific IgG1 (ng/mL)	20 730 ± 1637**	11 324 ± 2200	3412 ± 1244*

Only OVA-sensitized mice expressed OVA-specific antibodies. Comparison of IP adjuvant (Protocol 1A) vs. IP adjuvant-free (Protocol 1B) injection reveals an adjuvant related influence on OVA-specific IgG1 titres. Comparison of IP adjuvant-free (Protocol 1B) vs. SC adjuvant-free (Protocol 1C) sensitization reveals a site-specific influence on the generation of OVA-specific IgE and to a lesser extent, OVA-specific IgG1 titres.

* *P*<0.05

** *P*<0.01

*** *P*<0.001 vs. the IP adjuvant-free group.

Shown are mean ± SEM.

OVA, ovalbumin; IP, intraperitoneal; SC, subcutaneous.

Looking further into the OVA adjuvant-free model, we also tested whether varying the site of sensitization induced changes in allergic sensitization; the differences between i.p. and s.c. routes of delivery were examined using Protocol 1B and 1C (Fig. 1). Comparison of adjuvant-free i.p. and s.c. sensitization routes revealed that s.c. sensitization resulted in significantly higher OVA-specific IgE antibody production (see Table 1). Owing to the importance of IgE antibodies in allergy, Protocol 1C (the s.c. adjuvant-free protocol) was chosen for use in future experiments.

### Comparison of subcutaneous OVA adjuvant-free and intraperitoneal OVA adjuvant protocols using BALB/c and C57BL/6 mice

The second part of the study focused specifically on comparison of the phenotype generated by OVA adjuvant and adjuvant-free protocols. Since it was previously determined that the s.c. adjuvant-free model is most effective with Protocol 1 and the i.p. adjuvant model is most effective with Protocol 2 (Fig. 1) (data not shown), the most effective protocols were subsequently compared with each other. Additionally, since BALB/c and C57BL/6 mice represent the two most frequently used mouse strains in allergy research, comparisons were performed between these two mouse strains to examine possible differences in phenotype.

**Fig. 2:**
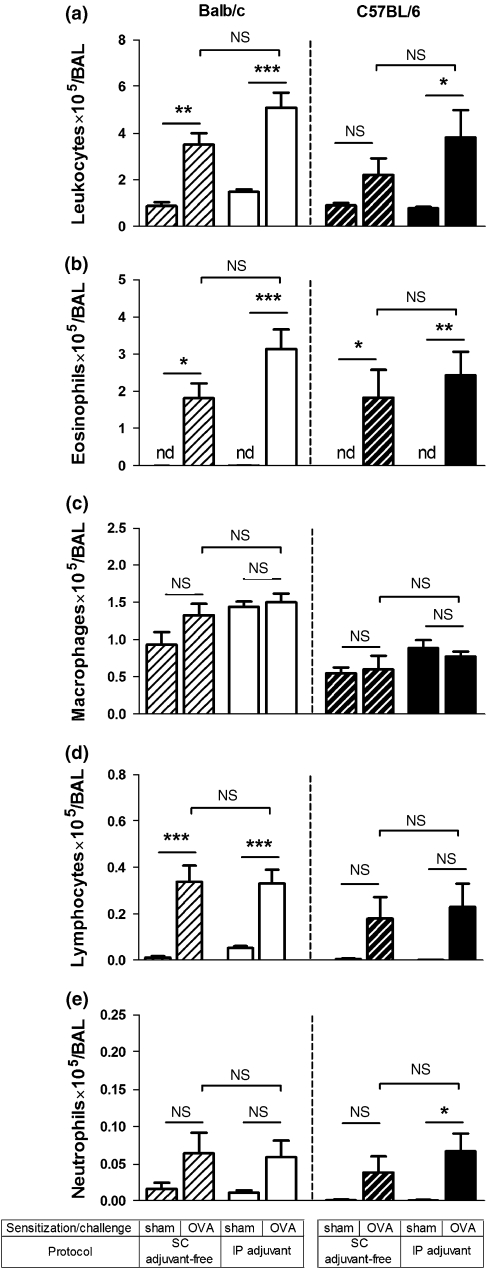
Total and differential cell counts in the BAL of BALB/c and C57BL/6 mice subjected to an OVA s.c. adjuvant-free (Protocol 1C) or OVA i.p. adjuvant protocol (Protocol 2A). (a) Leukocytes, (b) Eosinophils, (c) macrophages, (d) lymphocytes, and (e) neutrophils. Both protocols generated a comparable cell influx into the BAL fluids. BAL leukocytes and eosinophils were significantly increased between sham controls and the respective OVA-treated groups. Lymphocyte number significantly increased in BALB/c mice only. nd=not detectable, ns=not significant, *P<0.05, **P<0.01, ***P<0.001. Shown are mean ± SEM.

Both the s.c. adjuvant-free (Protocol 1C) and the i.p. adjuvant (Protocol 2A) protocols induced an equal amount of inflammatory cell infiltration into the BAL fluid in both mouse strains. The influx of eosinophils and lymphocytes, which were predominantly responsible for the increase in leukocyte cell numbers, also showed no differences upon comparison of the two protocols, shown in Fig. 2.

**Fig. 3:**
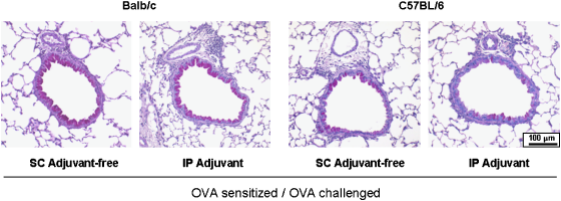
Representative PAS-stained lung histology sections of OVA-sensitized and -challenged BALB/c and C57BL/6 mice subjected to an s.c. adjuvant-free (Protocol 1C) or i.p. adjuvant protocol (Protocol 2A). The airways of BALB/c mice contained significantly more PAS-stained mucus-producing goblet cells than C57BL/6 mice regardless of the protocol used.

Observation of HE-stained lung sections from OVA-sensitized and -challenged animals demonstrated that both s.c. adjuvant-free (Protocol 1C) and i.p. adjuvant (Protocol 2A) protocols had similar lung histology and perivascular inflammation regardless of the strain (data not shown). Comparison of PAS-stained lungs between mouse strains revealed that regardless of the protocol used, OVA-sensitized and -challenged BALB/c mice had larger numbers of mucus-producing goblet cells than C57BL/6 animals. C57BL/6 mice exhibited less mucus production compared with BALB/c mice regardless of the protocol used (Fig. 3).

**Fig. 4:**
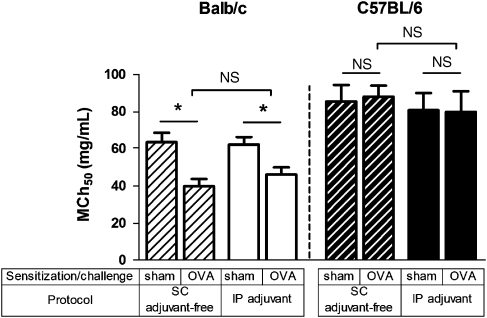
Airway reactivity to methacholine of BALB/c and C57BL/6 mice subjected to either s.c. adjuvant-free (Protocol 1C) or i.p. adjuvant (Protocol 2A) protocols using the antigen OVA. Both protocols led to comparable extents of airway reactivity in each respective mouse strain. In BALB/c mice, significant differences were observed between sham and OVA-sensitized groups, indicating the induction of airway hyperreactivity. This data also demonstrate the difficulty of achieving airway reactivity in the C57BL/6 strain. Airway responsiveness to methacholine was analysed by head-out body plethysmography 24 h after the last challenge. ns=not significant, *P<0.05. Shown are mean ± SEM.

As shown in Fig. 4, both s.c. adjuvant-free (Protocol 1C) and i.p. adjuvant (Protocol 2A) protocols used in this study had the ability to induce a significant increase in airway reactivity to methacholine in BALB/c mice compared with their sham-sensitized counterparts. In contrast, no differences in airway reactivity were observed in C57BL/6 mice regardless of the protocol used. Strain comparison indicated that the BALB/c mice had significantly higher airway reactivity than C57BL/6 mice (*P*<0.001).

As expected, observation of OVA-specific antibody titres from OVA-sensitized and -challenged mice demonstrated that the s.c. adjuvant-free protocol (Protocol 1C) resulted in significantly higher OVA-specific IgE and significantly lower OVA-specific IgG1 levels than the i.p. adjuvant protocol (Protocol 2A). Analysis of protocol vs. strain indicated that the protocol had a strong effect on both OVA-specific IgG1 (*P*<0.001) and OVA-specific IgE production (*P*<0.001). This is in agreement with the previous investigation of antibody levels in relation to adjuvant use and site of injection, seen in Table 1.

Interestingly, assessment of strain differences revealed that in OVA-sensitized mice, the BALB/c strain consistently produced higher OVA-specific IgE antibody titres than the C57BL/6 strain regardless of whether the adjuvant Protocol 2A (42 ± 7 vs. 6 ± 2 ng/mL, respectively, *P*<0.001) or adjuvant-free Protocol 1C (95 ± 11 vs. 24 ± 8 ng/mL, respectively, *P*<0.001) was used. Also in sensitized mice using the s.c. adjuvant-free Protocol 1C, BALB/c mice expressed higher OVA-specific IgG1 titres than C57BL/6 mice (2546 ± 434 vs. 337 ± 91 ng/mL, respectively, *P*<0.01). This trend was not observed when using the i.p. adjuvant Protocol 2A; both BALB/c and C57BL/6 mice expressed equivalent levels of OVA-specific IgG1 (20 410 ± 2663 vs. 26 010 ± 4258 ng/mL, respectively).

**Fig. 5:**
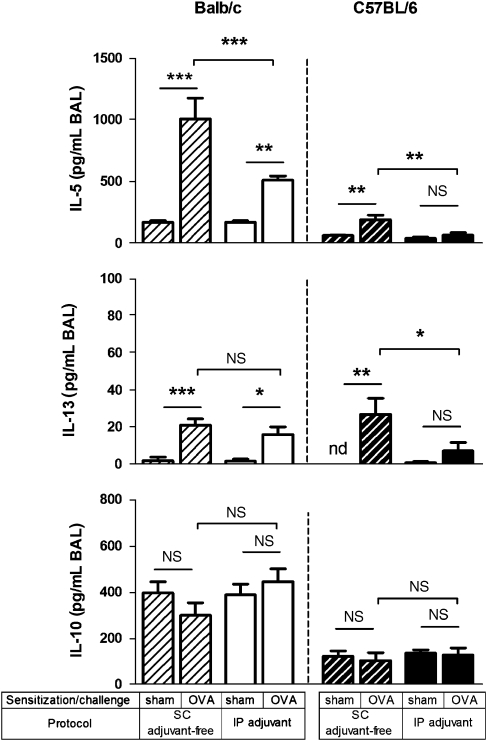
Cytokine concentrations measured in the BAL fluid of BALB/c and C57BL/6 mice using s.c. adjuvant-free (Protocol 1C) and i.p. adjuvant (Protocol 2A) models with the antigen OVA. Analysis illustrates that the s.c. adjuvant-free protocol generated greater levels of IL-5 than the i.p. adjuvant protocol. No differences were observed in IL-13 or IL-10. Further comparison of strains in both protocols demonstrates that BALB/c mice consistently produce higher levels of IL-5 and IL-10 than C57BL/6 mice. ns=not significant, *P<0.05, **P<0.01, ***P<0.001. Shown are mean ± SEM.

Cytokine analysis (Fig. 5) showed that in both strains of mice, the s.c. adjuvant-free protocol (Protocol 1C) induced higher BAL fluid IL-5 concentrations than the i.p. adjuvant protocol (Protocol 2A). Comparison of strain and protocol indicated that protocol type affected BAL fluid IL-13 levels (*P*<0.05) and showed a very significant influence of mouse strain on BAL fluid IL-10 levels (*P*<0.001). IFN-γ and TNF-α were not measurable in the BAL fluid of any of the samples tested.

### The subcutaneous adjuvant-free protocol functions with an alternative antigen

**Fig. 6:**
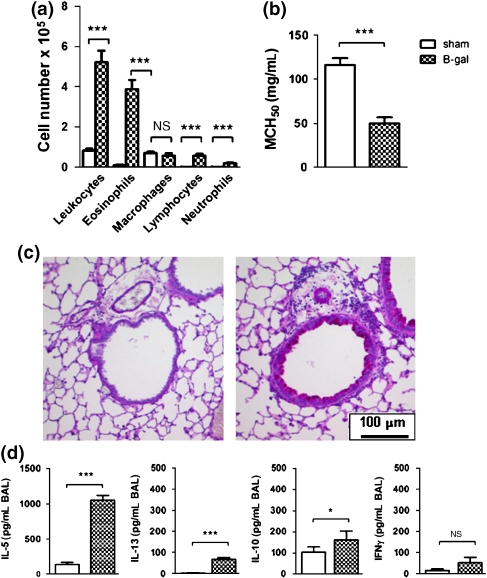
The airway inflammation phenotype generated by the s.c. adjuvant-free protocol utilizing the alternative antigen β-gal and i.n. challenge in BALB/c mice (Protocol 1D). β-Gal-sensitized and -challenged animals showed (a) an increased cell influx into the BAL fluid, (b) increased airway reactivity, (c) goblet cell hyperplasia in the lung and (d) significantly higher levels of BAL fluid IL-5, IL-13 and IL-10 than sham treated controls. ns=not significant, *P<0.05, ***P<0.001. Shown are mean ± SEM.

To demonstrate the versatility of the s.c. adjuvant-free protocol for use with antigens alternative to OVA, BALB/c mice were s.c. sensitized and i.n. challenged with β-gal using Protocol 1D (Fig. 1). Figure 6 illustrates (A) increases in BAL fluid leukocytes, eosinophils and lymphocytes, (B) induction of airway hyperreactivity, (C) goblet cell hyperplasia in the lung histology and (D) higher levels of BAL fluid IL-5, IL-13 and IL-10 in the β-gal-sensitized and -challenged mice compared with control animals. No differences were seen in IFN-g and TNF-α was not measurable in the BAL fluid. β-gal-specific IgE and IgG1 titres were also induced in β-gal-sensitized and challenged animals (data not shown).

### Intraperitoneal application of the adjuvant aluminum hydroxide causes severe stress in mice

**Fig. 7:**
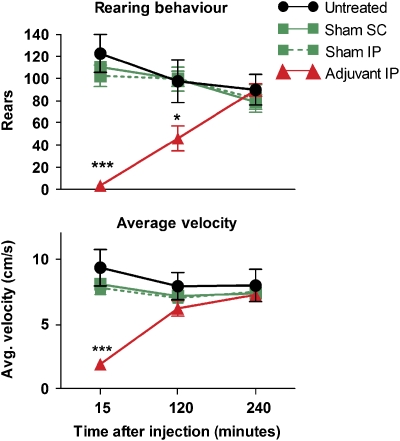
Behavioural tests measuring the average velocity and rearing in untreated, sham-injected and adjuvant-injected mice. Mice were either left untreated, injected with sham PBS (i.p. or s.c.) or adjuvant (i.p.+alum). After treatment, animals were allowed a 15-, 120- or 240-min rest period, and then subjected to open-field testing for 15 min. Black circles indicate the untreated group (no injection), green squares indicate mice sham injected either s.c. (solid line) or i.p. (dashed line), and red triangles indicate mice injected with i.p. with the adjuvant alum. *P<0.05, ***P<0.001 vs. untreated. Shown are mean ± SEM.

In the standardized open-field testing conducted, it was demonstrated that mice treated i.p. with the adjuvant alum displayed significantly decreased average velocity (movement) and rearing (awareness of surroundings) 15 min after treatment. This distress resolved within 2 or 4 h, respectively, Fig. 7. No differences were observed in the rearing or average velocity between untreated (non-injected) and sham-injected (i.p. or s.c.) groups. These data clearly demonstrate that injection with alum causes significant distress in mice up to 4 h after treatment.

## Discussion

Research investigating the underlying mechanisms of human disease, as well as the initial testing of therapeutic agents, depends heavily on animal models. Illustrating this, murine models are commonly used in asthma research and many different methods are used to generate acute allergic airway inflammation. On the whole, protocol selection and choice of mouse strain are paramount to the study design and it is desirable to mimic the natural physiology as closely as possible. In contrast to this, the most frequently used method to sensitize mice to allergen utilizes the non-physiological adjuvant alum to promote the development of a TH2-driven immunological response [27, 28].

Although alum is regularly used in mouse models of experimental asthma, its mechanism of action has only recently begun to be elucidated. Phagocytosis of alum plus antigen has been shown to result in phagosomal destabilization [29, 30], activation of the NALP3 inflammasome and production of pro-inflammatory cytokines such as IL-1β, IL-18 and IL-33 [3–6]. In addition to this, uric acid release is implicated in the mechanism of alum-mediated immunity via dendritic cell maturation [8, 9, 31] and co-stimulatory molecule expression [7]. How these mechanisms act to enhance humoral immunity is presently a topic of intense study.

Regardless of the mechanism by which alum acts as an adjuvant, there is a large body of evidence in the literature demonstrating that adjuvant is not necessary for the induction of murine experimental asthma [13–15, 32–35]. Although these protocols all produce acute allergic lung inflammation, there is a strong need for a head-to-head comparison of adjuvant and adjuvant-free protocols that specifically deduces similarities and differences in the phenotype. In order to compare adjuvant and adjuvant-free experimental asthma phenotypes, we first optimized an adjuvant-free protocol by initially testing several parameters including variation in the route of sensitization, length and time of aerosol challenge and antigen concentration. From these experiments, a simple OVA s.c. adjuvant-free protocol was produced. Use of this protocol eliminates artefacts that may result from alum use.

With the use of an adjuvant-free protocol, it is speculated that additional danger signals, such as LPS content in the OVA preparation, may be required to facilitate the sensitization process [36]. Indeed, Eisenbarth et al. [37] demonstrated that i.n. sensitization in the absence of LPS resulted in tolerance rather than sensitization. In contrast to this, recent experiments utilizing the s.c. adjuvant-free model described here with either LPS-containing or LPS-free OVA demonstrated that, regardless of LPS presence, similar experimental asthma phenotypes were generated (data not shown). This topic has raised interesting questions regarding the mechanism of sensitization using a parenteral (s.c.) adjuvant-free protocol and is currently the subject of further investigation.

Subsequent comparisons of the new OVA s.c. adjuvant-free protocol with an OVA i.p. adjuvant protocol frequently used in the literature demonstrated that with the exception of increased IL-5 production and differences in OVA-specific antibody titres, the s.c. adjuvant-free protocol produces an allergic airway inflammation phenotype comparable with the standardized i.p. adjuvant protocol. As it is well known that alum participates in the generation of humoral immunity [27], the differences in antibody titres can be easily explained. What is interesting about these findings is that alum appears to contribute solely to the generation of OVA-specific IgG1 antibodies as OVA-specific IgE titres were higher in the adjuvant-free protocol.

Comparison of the inflammatory phenotype generated by the i.p. adjuvant and s.c. adjuvant-free protocols in different strains of mice supports the notion that the choice of the mouse strain is a crucial aspect for experimental design. With regard to OVA-induced airway inflammation, we demonstrated that the magnitude of immune response with respect to goblet cell hyperplasia, airway reactivity, BAL fluid IL-5, IL-10 and serum OVA-specific IgE production is significantly lower in C57BL/6 mice than in BALB/c. These findings were similar regardless of whether an i.p. adjuvant or an s.c. adjuvant-free protocol was used. These findings support many others in the literature that have found lower acute inflammatory responses in C57BL/6 mice [25, 38–40].

During use of the i.p. adjuvant protocol, abnormal mouse behaviour was observed beginning 15 min after OVA alum injection. To investigate this further, we sought to quantify the effect of alum on mice using standardized, quantitative behavioural analysis. Open-field testing, which is used to assess anxiety, alertness, locomotor activity and exploration [26], revealed the novel finding that alum causes severe distress in mice for up to 4 h after i.p. injection. The direct cause of distress after alum injection is presently unknown; however, evidence regarding alum mechanisms suggests that a likely cause may be due to the induction of tissue necrosis [30] and/or a rapid local inflammatory response [8].

In conclusion, this research has demonstrated that the improved OVA s.c. adjuvant-free protocol used in this study generates a phenotype similar to the standard OVA i.p. adjuvant protocol used in the majority of studies investigating mechanisms of respiratory allergy in mice. For researchers with concerns about the non-physiological nature of alum, we demonstrate that the s.c. adjuvant-free model is an excellent alternative to adjuvant use.
